# Real‐world treatment and outcomes of patients with metastatic BRAF mutant colorectal cancer

**DOI:** 10.1002/cam4.5783

**Published:** 2023-03-13

**Authors:** Ting Xu, Jian Li, Zhenghang Wang, Xiaotian Zhang, Jun Zhou, Zhihao Lu, Lin Shen, Xicheng Wang

**Affiliations:** ^1^ Department of Gastrointestinal Oncology, Key Laboratory of Carcinogenesis and Translational Research (Ministry of Education) Peking University Cancer Hospital & Institute Beijing China

**Keywords:** *BRAF* mutation, intensive chemotherapy, metastatic colorectal cancer, predictive model, prognostic markers

## Abstract

**Background:**

*BRAF* mutation occurs in 5%–10% of metastatic colorectal cancers (mCRCs). Patients with *BRAF* mutant mCRC exhibit a specific metastatic pattern and poor prognosis. Survival outcomes are heterogeneous in cases of mCRC with a *BRAF* mutation. The optimal first‐line therapy is still controversial.

**Methods:**

We retrospectively reviewed the medical records of patients with mCRC between June 2010 and December 2021. Clinicopathologic characteristics, treatment and *BRAF* mutation testing results were collected. Patients with a BRAF mutation were included. Kaplan–Meier methods and log‐rank tests were used to analyze and compare survival. Cox proportional hazards regression was used to establish the predictive nomogram model.

**Results:**

Of the 4475 mCRC, 261 have a *BRAF* mutation, including 240 V600E and 21 non‐V600E mutants. The median overall survival (OS) was 18.2 months in the *BRAF* V600E mutant group versus 38.0 months in the non‐V600E mutant group (*p* = 0.022). ECOG score, tumor differentiation, liver metastasis, bone metastasis and primary tumor resection were independent prognostic factors for the OS of *BRAF* V600E mutant mCRC. A nomogram model was established using these factors. The median OS was 39.3 m, 18.2 m and 10.7 m for the low‐risk, intermediate‐risk and high‐risk groups defined by this model, respectively (*p* < 0.0001). Patients who received first‐line triplet chemotherapy ± bevacizumab had comparable progression free survival (PFS) and OS compared with those treated with doublets ± bevacizumab.

**Conclusion:**

*BRAF* V600E mutant mCRCs exhibit unfavorable and heterogeneous prognosis. The first‐line intensive chemotherapy did not confer a marked impact on the PFS and OS.

## Graphical abstract

Metastatic CRCs with BRAF mutation exhibit heterogeneous clinical characteristics and prognosis. A more optimized first‐line therapy is in urgent need for these patients.

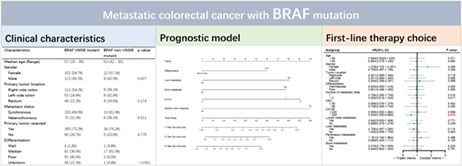

## 1 BACKGROUND

Colorectal cancer (CRC) is the third most common cancer and the leading cause of cancer‐related mortality worldwide.^1^ Despite abundant improvement in cancer research and drug development, patients with metastatic disease still have poor outcomes. With the increase in understanding of genetic drivers of tumor biology, it is recognized that CRC is a heterogeneous disease with different molecular characteristics and distinct prognoses.

The *BRAF* gene, one of the key members in the mitogen‐activated protein kinase (MAPK) pathway, is mutated in 5%–10% of metastatic colorectal cancers.^2^ Aberrant activation of serine/threonine kinases caused by oncogenic *BRAF* mutation could constitutively activate the MAPK signaling pathway and drive tumor proliferation and antiapoptotic behavior.^3^ With the evolving technology for mutational and functional evaluation, various *BRAF* mutations have been identified and studied. BRAF mutations can be classified into 3 classes according to their influence on kinase activity.^4^ Class 1, that is, the *BRAF* V600E mutation, accounts for approximately 90% of all *BRAF* mutations and its kinase activity is hyperactivated in an RAS‐independent manner. Class 2 mutations, with intermediately elevated kinase activity, normally occur in codons 601 and 597. Mutations in codons 594 and 596 are assigned to class 3, which result in reduced B‐RAF kinase activity in vitro.^4^, ^5^, ^6^ The clinical characteristics and prognosis differed significantly by BRAF mutations in CRC. Specifically, compared with those with BRAF V600E mutation, patients with BRAF non‐V600E mutations were younger and less likely to be female. And BRAF V600E mutant CRCs were more likely to have right‐side colon tumor, lymph node and peritoneal metastases.^7^, ^8^

The reason why *BRAF* V600E mutant mCRC is of particular concern in clinical practice is that patients with *BRAF* mutant mCRC had a prominently unfavorable prognosis in comparison with *BRAF* wild‐type and *BRAF* non‐V600E mutant mCRC.^2^ The *BRAF* V600E mutation was associated with specific clinical features in CRC, including elderly age, female sex, right‐side colon location, poor differentiation, mucinous histology, and lymph node and peritoneal metastasis.^9^ As reported in previous studies, the median overall survival of *BRAF* V600E mutant mCRC ranged from 9.8 to 18.2 months, which was significantly shorter than that of *BRAF* wild‐type patients.^3^, ^10^, ^11^ On the one hand, they lack sensitivity to chemotherapy, and on the other hand, *BRAF* mutant mCRC patients are less likely to receive subsequent treatment after first‐line therapy progression because of rapid deterioration.^9^

The optimal first‐line therapy for *BRAF* V600E mutant mCRC is still controversial. In the phase III TRIBE study, the median overall survival (OS) of *BRAF* mutant patients treated with FOLFOXIRI plus bevacizumab was 19.0 months, which was superior to the FOLFIRI plus bevacizumab regimen.^12^ Although limited *BRAF* mutant patients were enrolled, the results suggested that FOLFOXIRI plus bevacizumab might be a promising first‐line choice. A recently published meta‐analysis of five randomized clinical trials compared the efficacy of FOLFOXIRI plus bevacizumab and doublets plus bevacizumab. However, within the *BRAF* mutant subgroup (*n* = 115), the median OS was comparable between the two treatment groups (hazard ratio = 1.14 [95% CI 0.75–1.73]).^13^

Most of the treatment evidence of *BRAF* mutant mCRC was derived from the subgroup analysis of clinical studies due to the rarity of this population. However, these results inevitably are biased due to the strict selection criteria of each trial. In the present study, we summarized the treatment and outcomes of *BRAF* mutant mCRC with the aim of exploring the prognostic stratification and efficacy of first‐line therapy in real‐world settings.

## 2 METHODS

### 2.1 Participants and data collection

This was a retrospective cohort study. We reviewed the medical records of all metastatic colorectal cancer patients diagnosed at Peking University Cancer Hospital between June 2010 and December 2021. Patients with documented *BRAF* gene mutations were enrolled in this study. Demographic information, clinical information and treatment regimens were collected from the electronic medical records when metastatic disease was confirmed. Survival states were retrieved by telephone follow‐up. *RAS* and *BRAF* gene mutational status were confirmed by Sanger sequencing or next‐generation sequencing using the tumor tissue.

### 2.2 Statistical analysis

Chi‐square tests were used to compare the categorical characteristics between different groups. OS was defined as the period from the diagnosis of metastatic disease to the date of death due to any reason. Survival data were estimated using the Kaplan–Meier method. Log‐rank tests were used to compare the PFS and OS of distinct groups. Independent prognostic factors were selected by multivariate Cox regression analysis and were further retained in the prognostic model. The model performance was evaluated by a calibration curve and Harrell C index. Statistical analyses were performed using GraphPad Prism software 8.3.0 (GraphPad Software, Inc.) and R software, version 4.1. All reported *p* values were based on two‐tailed testing, and a *p* value below 0.05 was deemed statistically significant.

## 3 RESULTS

### 3.1 Patient Characteristics

Of the 4475 metastatic CRC patients with documented *BRAF* gene testing results, 261 were reported to have a *BRAF* mutation between June 2010 and December 2021. Fifteen patients who lacked sufficient clinical information or did not receive any systemic treatment were excluded from further analyses. Of the remaining 246 *BRAF* mutant mCRCs, *BRAF* V600E mutation accounted for 91.5% (*n* = 235) of all cases. The *BRAF* non‐V600E mutations included 6 class 2 mutations (3 p. G469A, 2 p. K601E, and 1 p. V504_R506dup), 9 class 3 mutations (8 p. D594H/G and 1 p. G595 L) and 6 unclassified mutations (1 p. S36A, 1 p. G672R, 1 p. Q201P, 1 p. P453A, 1 p.599_600insAGA and 1 c.1799_1801del).

Among the *BRAF* V600E mutant mCRC patients, 54.7% were female. The median age at metastatic disease was 57 (range: 19–88). Regarding the primary tumor location, the *BRAF* V600E mutation was more commonly seen in right‐side colon cancer (54.2%), while left‐side and rectal tumors were observed in 24.4% and 21.3% of patients, respectively. Most tumors were poorly or moderately differentiated, and 40 (17.8%) tumors had mucinous carcinoma or signet‐ring cell components. A total of 68.9% of patients had metastatic disease at the first diagnosis. A total of 155 (68.9%) mCRC patients had multiple organ metastasis. Distant lymph node metastasis was reported in 136 (60.4%) patients, and liver, peritoneum, pulmonary and bone metastasis occurred in 50.7%, 47.6%, 25.3% and 9.3% of patients, respectively. Microsatellite status was available for 186 cases, of which 11 (5.9%) were microsatellite instable, and 175 (94.1%) were microsatellite stable. None of the *BRAF* V600E mutant mCRCs had *KRAS* mutations.

Of the 21 *BRAF* non‐V600E mutant mCRCs, the median age was 63 years (range: 42–85). Compared with the *BRAF* V600E mutation, the non‐V600E mutation was associated with well/median differentiation (*p* < 0.001), single organ metastasis (*p* = 0.047), and less lymph node (*p* = 0.047) and peritoneum (*p* = 0.037) involvement. Furthermore, non‐V600E mutant mCRC was more likely to originate from the left colon, although statistical significance was not reached (*p* = 0.174). *KRAS* mutation was detected in 3 (14.3%) patients who all harbored a class 2 *BRAF* gene mutation. Detailed demographic and clinical characteristics of V600E and non‐V600E mutant mCRC patients are summarized in Table 1.

**TABLE 1:** Patient characteristics.

Characteristics	BRAF V600E mutant	BRAF non‐V600E mutant	*p* value
Median age (Range)	57 (19–88)	63 (42–85)	
Gender			0.827
Female	102 (54.7%)	12 (57.1%)	
Male	123 (45.3%)	9 (42.9%)	
Primary tumor location			0.174
Right‐side colon	122 (54.2%)	8 (38.1%)	
Left‐side colon	55 (24.4%)	9 (42.9%)	
Rectum	48 (21.3%)	4 (19.0%)	
Metastasis status			0.511
Synchronous	155 (68.9%)	13 (61.9%)	
Heterochronous	70 (31.3%)	8 (38.1%)	
Primary tumor resected			0.776
Yes	165 (73.3%)	16 (76.2%)	
No	60 (26.7%)	5 (23.8%)	
Differentiation			<0.001
Well	4 (1.8%)	1 (4.8%)	
Median	82 (36.4%)	17 (81.0%)	
Poor	91 (40.4%)	2 (9.5%)	
Unknown	48 (21.3%)	1 (4.8%)	
Any component of Mucinous histology or Signet‐ring cell carcinoma			0.030
Yes	40 (17.8%)	0 (0)	
No	185 (82.2%)	21 (100.0%)	
Number of metastatic sites			0.047
1	70 (31.1%)	11 (52.4%)	
≥2	155 (68.9%)	10 (47.6%)	
Metastatic organ			
Lymph node	136 (60.4%)	8 (38.1%)	0.047
Liver	114 (50.7%)	11 (52.4%)	0.0881
Lung	57 (25.3%)	6 (28.6%)	0.745
Peritoneum	107 (47.6%)	5 (23.8%)	0.037
Bone	21 (9.3%)	3 (14.3%)	0.464
Others	50 (22.2%)	3 (14.3%)	0.398
ECGO PS score			
0	83 (36.9%)	11 (52.4%)	0.400
1	126 (56.0%)	10 (47.6%)	
≥2	13 (5.8%)	0 (0)	
Unknown	3 (1.3%)	0 (0)	
Microsatellite status			0.323
pMMR/MSS	175 (77.8%)	19 (90.5%)	
dMMR/MSI‐H	11 (4.9%)	1 (4.8%)	
Unknown	39 (17.3%)	1 (4.8%)	
KRAS status			0.001
Wild type	225 (100.0%)	18 (85.7%)	
Mutant	0 (0)	3 (14.3%)	

### 3.2 Prognosis of *BRAF* mutant metastatic colorectal cancer

**FIGURE 1:**
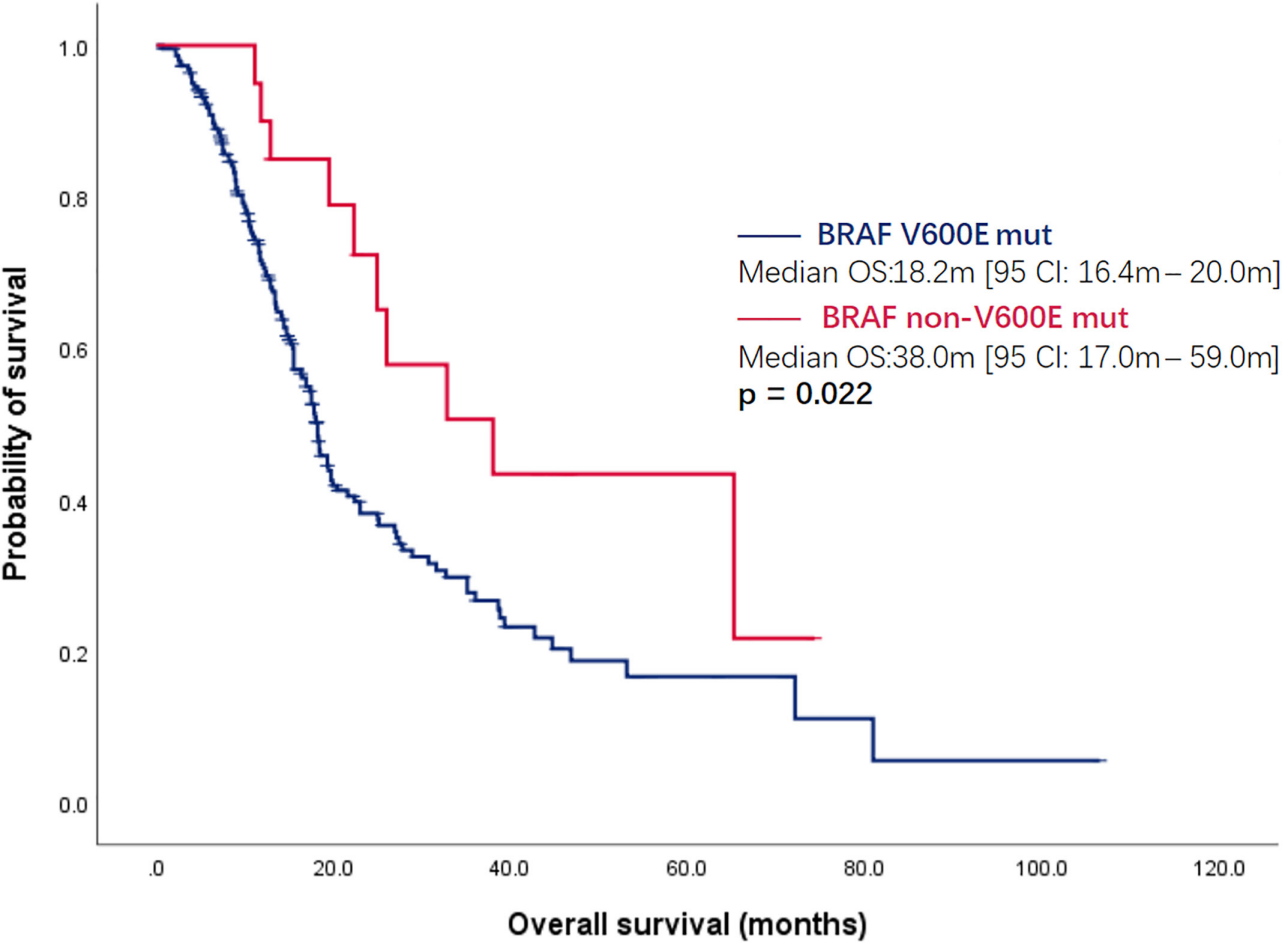
Overall survival of BRAF V600E mutant and non‐V600E mutant mCRC.

After a median follow‐up of 33.4 months (95% CI: 26.1–40.7 m), 138 *BRAF* V600E mutant and 10 non‐V600E mutant mCRC patients died. The median OS of *BRAF* V600E mutant mCRC was significantly worse than that of the non‐V600E mutant population (18.2 m [95% CI: 16.4–20.0 m] vs. 38.0 m [95% CI: 17.0–59.0 m], *p* = 0.022) (Figure 1). At the same time, significant differences in prognosis were observed among these patients. In our cohort, we found that 37.8% of patients survived over 24 months, and 26.7% of patients survived less than 12 months. Thus, we aimed to establish an efficient clinical parameter‐based model to predict the outcomes of *BRAF* V600E mutant mCRC.

First, univariate analysis was used to screen the potential prognostic clinical characteristics of *BRAF* V600E mutant mCRC (Table 2). Male sex (*p* = 0.047), poor differentiation (*p* = 0.017), unresected primary tumor (*p* < 0.0001), and high ECOG score (*p* < 0.0001) were associated with unfavorable prognosis. Furthermore, patients with multiple organ metastasis (*p* < 0.0001), distant lymph node metastasis (*p* = 0.003), liver metastasis (*p* < 0.0001) and bone metastasis (*p* = 0.017) had remarkably shorter OS in our cohort. OS was significantly shorter in patients with elevated levels of CEA (*p* = 0.001) and CA19‐9 (*p* < 0.0001). BRAF V600E mutant mCRC originating from the left‐side colon seemed to have superior overall survival compared with right‐side and rectal tumors, although statistical significance was not reached (median OS for left‐side, right‐side and rectal cancer: 20.4 m, 16.9 m, and 16.4 m, *p* = 0.296). Microsatellite status was not related to overall survival.

**TABLE 2:** Univariate and Multivariate analysis of overall survival.

Characteristics	Median OS (m)	Univariate analysis		Multivariate analysis	
		HR [95%CI]	*p* value	HR [95%CI]	*p* value
Age					
<40	16.4 [12.2–20.6]	Reference	0.298		
≥40, <60	19.3 [17.4–21.2]	0.699 [0.433–1.127]			
≥60	18.2 [16.7–19.7]	0.721 [0.449–1.155]			
Gender					
Male	21.6 [15.0–28.2]	**Reference**	**0.056**	1.188 [0.747–1.891]	0.467
Female	15.5 [13.1–17.9]	**1.409 [0.990–1.951]**			
Primary tumor location					
Right‐side	16.9 [14.7–19.1]	Reference	0.344		
Left‐side	20.4 [14.1–26.7]	0.751 [0.497–1.135]			
Rectum	16.4 [10.2–22.6]	1.032 [0.661–1.611]			
Metastasis status					
Synchronous	17.8 [15.5–20.1]	Reference	0.196		
Heterochronic	22.4 [12.2–32.6]	0.781 [0.536–1.137]			
Primary tumor resected					
No	11.7 [7.9–15.5]	**Reference**	**<0.0001**	**0.486 [0.299–0.789]**	**0.004**
Yes	21.6 [14.9–28.3]	**0.366 [0.252–0.532]**			
Differentiation					
Poor	15.5 [12.8–18.2]	**Reference**	**0.020**	**0.672 [0.406–0.969]**	**0.036**
Median/Well	27.1 [18.4–35.8]	**0.634 [0.430–0.935]**			
Any component of Mucinous histology or Signet‐ring cell carcinoma					
No	18.2 [16.9–19.5]	Reference	0.760		
Yes	16.9 [6.1–27.7]	1.069 [0.696–1.643]			
Number of metastatic sites					
1	30.7 [23.2–38.2]	**Reference**	**<0.0001**	1.182 [0.636–2.198]	0.597
≥2	15.4 [13.3–17.6]	**2.299 [1.534–3.446]**			
Lymph node metastasis					
No	27.1 [18.7–35.5]	**Reference**	**0.005**	1.169 [0.691–1.977]	0.561
Yes	15.0 [12.5–17.5]	**1.673 [1.167–2.399]**			
Liver metastasis					
No	27.4 [18.8–36.0]	**Reference**	**<0.0001**	**2.000 [1.203–3.232]**	**0.007**
Yes	14.4 [12.3–16.5]	**2.327 [1.638–3.307]**			
Lung metastasis					
No	18.2 [16.3–20.1]	Reference	0.919		
Yes	18.0 [15.5–20.4]	1.020 [0.691–1.506]			
Peritoneum metastasis					
No	18.2 [15.4–30.0]	Reference	0.922		
Yes	18.2 [16.1–20.3]	0.983 [0.699–1.382]			
Bone metastasis					
No	19.3 [16.7–21.9]	**Reference**	**0.007**	**2.415 [1.147–5.086]**	**0.020**
Yes	14.4 [12.3–16.5]	**1.996 [1.193–3.340]**			
CEA					
<90	18.2 [15.8–20.6]	**Reference**	**0.001**	1.16 [0.637–2.118]	0.626
≥90	12.3 [5.5–19.1]	**2.133 [1.341–3.393]**			
CA199					
<200	19.3 [13.3–25.3]	**Reference**	**<0.0001**	1.133 [0.803–1.598]	0.476
≥200, <6000	15.5 [12.3–18.7]	**1.567 [1.047–2.346]**			
≥6000	10.9 [2.8–19.0]	**3.928 [2.202–7.006]**			
ECOG PS					
0	30.7 [21.3–40.0]	**Reference**	**<0.0001**	**1.573 [1.079–2.294]**	**0.019**
1	16.9 [14.8–19.0]	**1.531 [1.020–2.298]**			
≥2	9.7 [6.3–13.1]	**3.359 [2.226–7.090]**			
Microsatellite status					
pMMR/MSS	18.5 [16.5–20.5]	Reference	0.235		
dMMR/MSI	53.1 [13.9–92.3]	0.607 [0.265–1.394]			

*p* values ＜ 0.05 are bolded.

**FIGURE 2:**
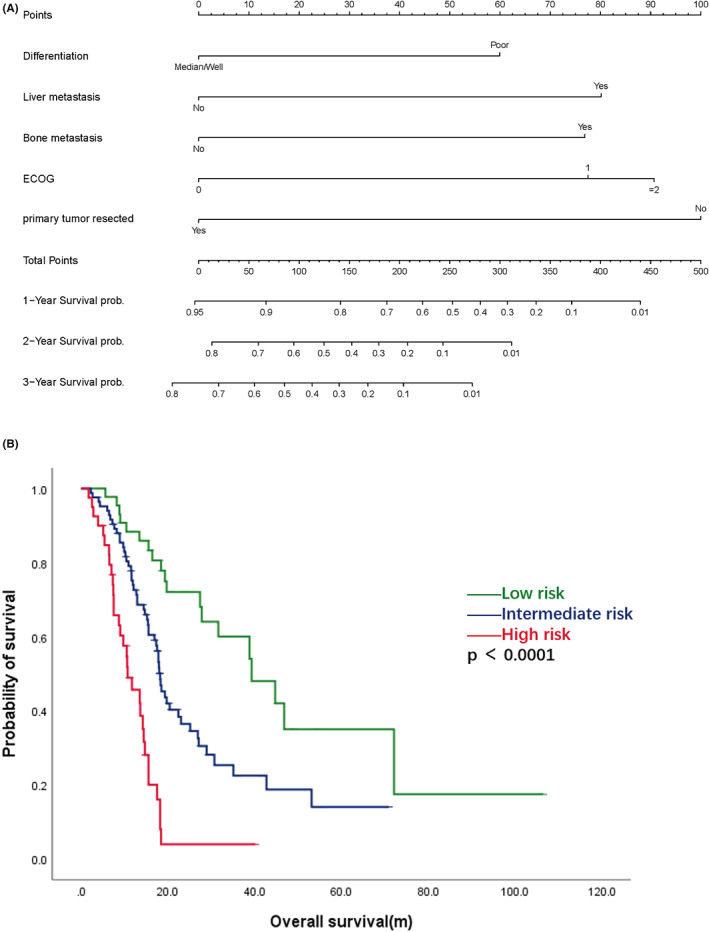
(A) Nomogram to predict the overall survival of BRAF V600E mutant mCRC. (B) Overall survival according to risk stratification: red line, high‐risk group, median OS was 10.7 m (95% CI: 8.7–14.7 m); blue‐line intermediate‐risk group, median OS was 18.2 m (95% CI: 16.9–25.2 m); green line, low‐risk group, median OS was 39.3 m (95% CI: 27.8–not reached).

In the multivariable analysis, tumor differentiation (*p* = 0.036), primary tumor resection (*p* = 0.004), ECOG score (*p* = 0.019), liver metastasis (*p* = 0.007) and bone metastasis (*p* = 0.020) maintained their prognostic effect on OS (Table 2). The five independent prognostic factors were integrated to develop a nomogram model to predict overall survival, as depicted in Figure 2A. The C‐index value of the model was 0.715 (95% CI: 0.661–0.769). Calibration curves indicated that the predicted 12, 24 and 36‐month survival rates were close to the true circumstance (Figure S1). We further classified BRAF V600E mutant mCRC into three risk categories according to their risk score in this model. The low‐, intermediate‐ and high‐risk cases accounted for 26.6%, 49.7% and 23.7% of the population, respectively. The median OS of the three groups was significantly different. For low‐risk patients, it was 39.3 m (95% CI: 27.8–not reached); for the intermediate‐risk group, it was 18.2 m (95% CI: 16.9–25.2 m); and for high‐risk patients, it was 10.7 m (95% CI: 8.7–14.7 m) (Figure 2B).

### 3.3 First‐line treatment and outcomes of BRAF V600E mutant mCRC

**FIGURE 3:**
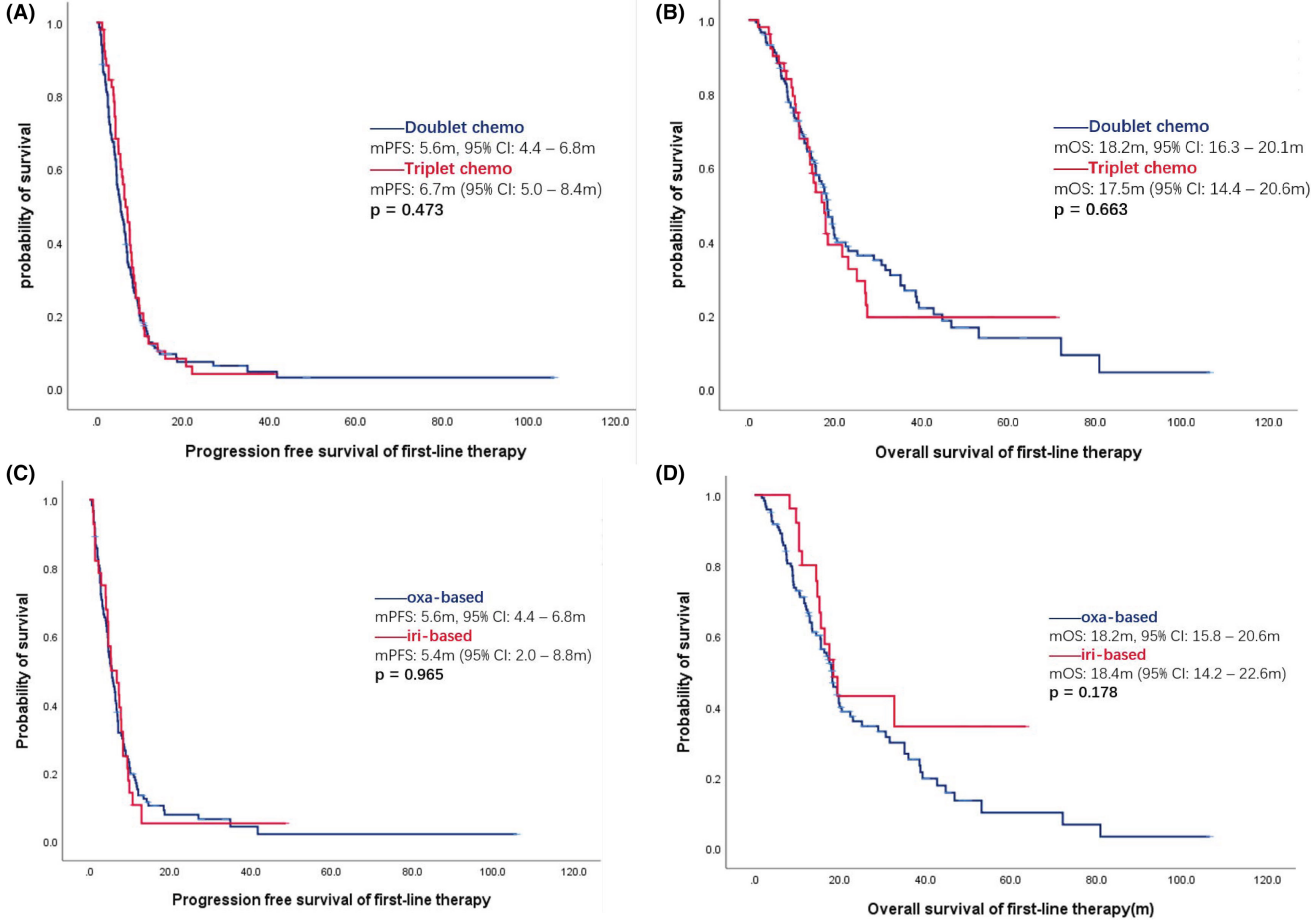
Progression‐free survival and overall survival following the first‐line therapy. (A) PFS of doublet (FOLFOX/FOLFIRI) and triplet (FOLFOXIRI) chemotherapy; (B) OS of doublet (FOLFOX/FOLFIRI) and triplet (FOLFOXIRI) chemotherapy; (C) PFS of oxaliplatin‐based and irinotecan‐based doublet chemotherapy; (D) OS of oxaliplatin‐based and irinotecan‐based doublet chemotherapy.

As expected, the PFS of first‐line therapy was longer in *BRAF* non‐V600E mutant mCRC cases than in V600E mutant mCRC cases (11.8 m vs. 6.4 m, *p* = 0.002). Of the 217 *BRAF* V600E mutant mCRCs with available first‐line treatment data, 52 patients accepted a triplet chemotherapy regimen (fluorouracil, oxaliplatin and irinotecan) ± bevacizumab; 152 patients were treated with doublet chemotherapy (fluorouracil plus oxaliplatin or irinotecan) ± bevacizumab (Table S1). The median PFS of the triplet regimen was 6.7 m (95% CI: 5.0–8.4 m), which was comparable to that of the doublet chemotherapy (5.6 m, 95% CI: 4.4–6.8 m) (*p* = 0.473) (Figure 3A). In addition, no significant difference was observed in OS between the two treatment groups (*p* = 0.663) (Figure 3B).

The inconsistent results from previous clinical trials comparing triplet and doublet regimens could arise from the different chemotherapeutic agents used in the control group. Thus, we further examined the impact of the chemo backbone on the efficacy of first‐line therapy. Of the 152 patients treated with chemotherapy doublets, 125 received an oxaliplatin‐containing regimen and 27 received an irinotecan‐containing regimen. No significant difference was observed either in PFS (HR = 1.010, 95% CI: 0.657–1.551, *p* = 0.965) or in OS (HR = 0.679, 95% CI: 0.385–1.198, *p* = 0.179) (Figure 3C,D).

**FIGURE 4:**
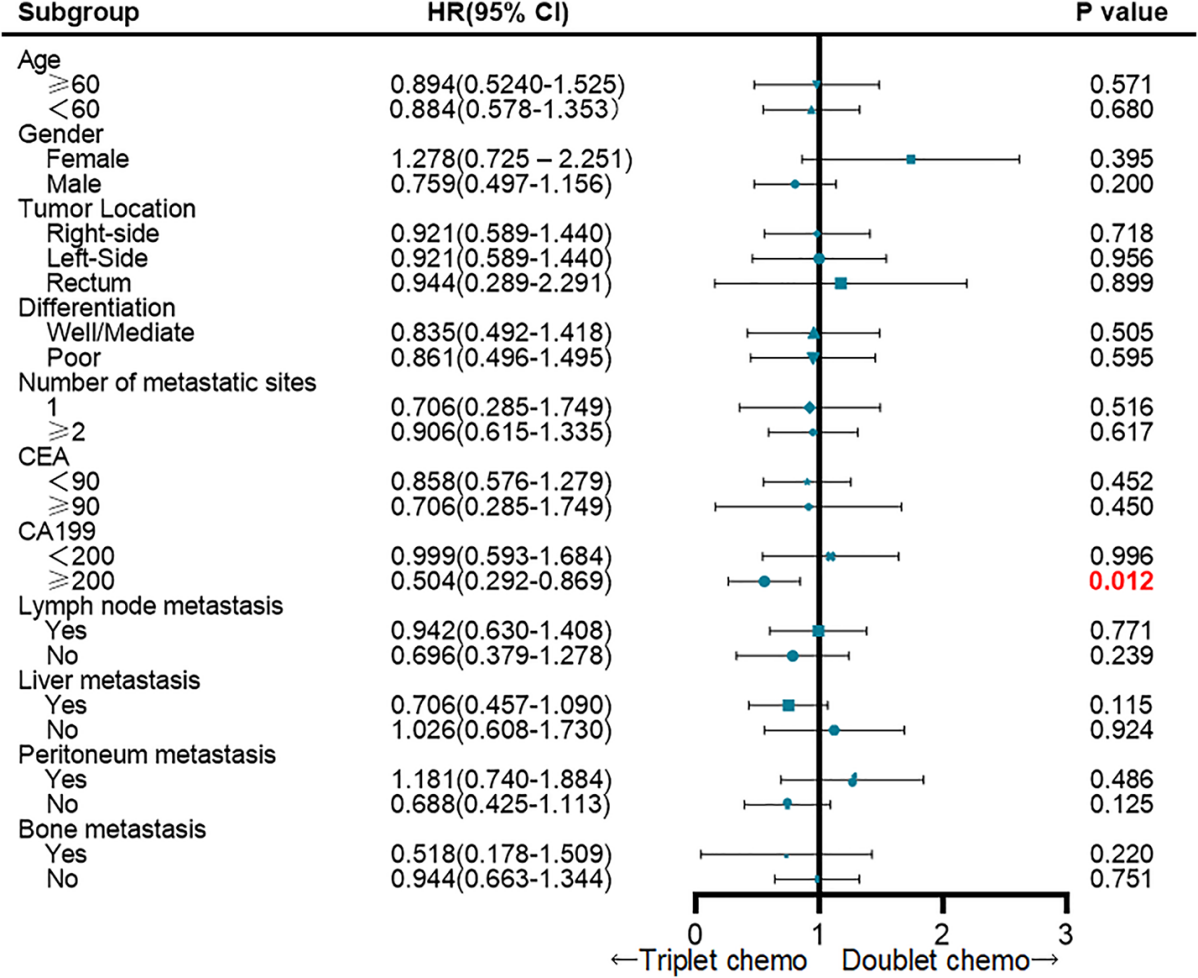
Treatment effect on progression‐free survival within the main clinical subgroups of doublet (FOLFOX/FOLFIRI) and triplet (FOLFOXIRI) first‐line chemotherapy.

In most of the subgroups, the treatment effects of doublet and triplet chemotherapy were similar in PFS (Figure 4), except that triplet chemotherapy could improve PFS in patients with elevated CA19‐9 levels (≥200 U/L at baseline) (HR = 0.504, 95% CI: 0.292–0.869, *p* = 0.012). The OS results in the CA19‐9 elevated subgroup exhibited a borderline advantage from the triplet regimen (HR = 0.570, 95% CI: 0.313–1.035, *p* = 0.057).

## 4 DISCUSSION

*BRAF* mutation in colorectal cancer has recently received increasing attention for its prognostic effects and targetable properties. Herein, we report one of the largest and the first Chinese cohort of *BRAF mutant* mCRC based on real‐world data. There are several findings of clinical significance from our study. First, we found differences in clinical characteristics between Chinese and western mCRC patients with *BRAF* mutation, including younger age of onset and low microsatellite instability frequency in our cohort. Second, our results show obvious heterogeneity in outcomes of *BRAF* V600E mutant mCRC, and a prognostic classifier according to baseline clinical parameters was able to validly predict the overall survival of these patients. Finally, commonly used first‐line chemotherapy regimens in clinical practice were compared in this study, and no significant therapeutic efficacy difference was observed in real‐world settings.

In prior studies, the *BRAF* V600E mutation in colorectal cancer was associated with an older age of onset, female sex, right‐side colon origination, poor differentiation and mucinous adenocarcinoma pathological phenotypes compared with *BRAF* wild‐type or *BRAF* non‐V600E mutant CRC.^9^ The metastatic pattern of *BRAF* V600E mutant mCRC usually involves multiple organs with a high frequency of distant lymph node and peritoneal metastasis. The clinical‐pathological characteristics of *BRAF* V600E mutant mCRC in our cohort were mostly consistent with previous results; however, several discrepancies were observed. In *BRAF* V600E mutant mCRC, the previously reported median age at metastatic disease was 64 to 66 years, and patients over 60 accounted for over 70%.^9^, ^14^ However, in our cohort, the median age of onset was 57 years, and 59.1% of patients were diagnosed before the age of 60. Furthermore, it was reported that approximately 20%–30% of *BRAF* V600E mutant CRCs display mismatch repair protein deficiency or microsatellite instability (MSI). The frequency decreased to 5.9% (11/186) in our cohort, which was similar to that of *BRAF* wild‐type CRC. The co‐occurrence of *BRAF* mutation and MSI was mainly due to an elevated level of CpG island methylator phenotype (CIMP) and MLH1 promoter methylation in *BRAF* mutant CRC.^15^ CIMP was also positively correlated with age of onset. The relatively early age of onset of our cohort and late‐stage disease may explain the low frequency of MSI in our results.

The *BRAF* V600E mutation is a well‐recognized unfavorable prognostic factor in mCRC.^2^ The overall survival of patients with *BRAF* V600E mutant mCRC was 18.2 months in our real‐world data, which was significantly shorter than that of *BRAF* non‐V600E mutant mCRC. The differences in OS could to some extent account for the imbalance in clinicopathological characteristics between the two groups. Indeed, the *BRAF* V600E mutation was associated with several unfavorable prognostic factors, including right‐side colon origination, poor differentiation and multiple organ metastasis. Nevertheless, the *BRAF* V600E mutation retained the negative effect on first line PFS (*p* = 0.003) and OS (*p* = 0.042) after multivariate survival analysis, which confirmed the high invasiveness and insensitivity to chemotherapy of CRC harboring the *BRAF* V600E mutation. It should be noticed that the overall survival of our cohort seems better than the data from earlier studies.^9^, ^10^ One crucial reason was the advent of novel treatment agents in recent years. In our cohort, 63.6% (7/11) MSI‐H patients received immunotherapy and 36.5% (64/175) MSS patients received BRAF inhibitor combinations during treatment.

In a post hoc analysis of 3 randomized trials of first‐line chemotherapy for mCRC, 231 *BRAF* V600E mutant mCRCs were included, and 24% of them survived over 24 months, although only doublet chemotherapy was used as the first‐line therapy.^9^ With the increasing attention to *BRAF* mutations in mCRC, the heterogeneity of the treatment outcomes and molecular characteristics comes to light. From a genetic perspective, *BRAF* mutant CRC could be classified into two groups according to Wnt pathway activation, including *APC* mutant ligand‐independent and *RNF43/RSPO* mutant ligand‐dependent subtypes.^16^ At the transcriptomic level, two classifications have been posed: the consensus molecular subtypes (CMSs) and the BRAF‐mutant (BM) subtype. Of the four CMS subgroups, CMS1 (immune) and CMS4 (mesenchymal) are enriched in BRAF V600E mutant CRC.^7^, ^17^ Also, in the BM system, BM1, characterized by *KRAS/AKT* pathway activation and epithelial‐mesenchymal transition, accounts for 30% of all BRAF V600E mutant CRC. BM2 subtype demonstrates deregulation of cell‐cycle checkpoints and better overall prognosis compared with BM1 subtype.^18^, ^19^ However, these classifications are technically demanding. Clinically, it is important to establish an easy‐to‐use prognostic stratification method for *BRAF* V600E mutant mCRC. First, the prognostic factors of whole mCRC, for example, right‐side tumor location, are not inapplicable for this specific population. This model could help oncologists and patients have a relatively accurate expectation of patient prognosis. Second, from the perspective of clinical practice, for those with low‐risk tumors, the life expectancy was similar to that of *BRAF* wild‐type patients. Intensified chemotherapy should not be used in these patients to avoid additional adverse events. In addition, oncologists could treat them more like *BRAF* wild‐type patients when making medical decisions, especially when considering locoregional therapies. Furthermore, this prognostic model could be considered a stratification factor in randomized controlled clinical trials to reduce confounding bias. Another two prognostic classifiers have been developed previously to stratify the overall survival and treatment efficacy of BRAF‐targeted therapy in BRAF V600E mutant mCRC. It is uniformly discovered in these models that surrogates of tumor load such as ECOG and CEA, and liver metastasis are significantly associated with the prognosis of BRAF V600E mutant CRC.^20^, ^21^

Considering the extremely aggressive property of *BRAF* V600E mutant mCRC, first‐line systemic antitumor therapy is of paramount significance for these patients to obtain rapid control of disease progression and provide opportunities for further treatment. Intensive chemotherapy combined with bevacizumab was previously thought to be a potentially optimal choice based on the impressive OS improvement of *BRAF* V600E mutant mCRC treated with FOLFOXIRI plus bevacizumab in the TRIBE trial (mOS: 19.0 m in the FOLFOXIRI plus bevacizumab group versus 10.7 m in the FOLFIRI plus bevacizumab group).^12^ However, in the TRIBE2 study, upfront FOLFOXIRI+ bevacizumab and reintroduction after progression did not exhibit superior treatment efficacy in terms of either first PFS (HR = 1.02, 95% CI: 0.61–1.71) or PFS2 (HR = 1.23, 95% CI: 0.72–2.09) over mFOLFOX + bevacizumab followed by FOLFIRI plus bevacizumab in the BRAF mutant subgroup (*n* = 66).^22^ A meta‐analysis published in 2020 collected 5 trials comparing FOLFOXIRI plus bevacizumab and doublets plus bevacizumab as the first‐line therapy in mCRC. A total of 115 *BRAF*‐mutated mCRCs were included, including 54 in the control group and 61 in the experimental group. The median OS of the two groups was 13.6 and 14.5 m, respectively (HR = 1.15, 95% CI 0.75–1.73), which indicated that there was no increased benefit from the intensified regimen.^13^ Similarly, our results also supported the notion that intensive chemotherapy should not be recommended as the optimal choice for all mCRCs with *BRAF* V600E mutation. Although not very robust, the subgroup analysis suggested that the CA199 level could be a surrogate of tumor burden to determine who might benefit from triplet chemotherapy.

Moreover, it should be noted that although PFS was not improved, triplet chemotherapy had a higher response rate than doublets in previous studies.^12^, ^13^ This result was important because remarkable tumor regression after systemic treatment might convert metastatic disease from unresectable to resectable. Liver‐only metastasis is relatively rare in patients with *BRAF* V600E mutation because of common lymph node and peritoneal involvement.^23^ The *BRAF* V600E mutation has been identified as a negative predictor of OS in colorectal liver metastasis resection in several studies. However, in a recent respective study, 105 liver‐limited CRLM patients with *BRAF* V600E mutation were enrolled. The OS was significantly prolonged in patients who received liver resection compared with those who did not (34 m vs. 10.6 m, *p* < 0.0001).^24^ Thus, the high response rate of triplet chemotherapy should be taken into account when choosing the systemic therapy for *BRAF* V600E mutant mCRC with surgery potential to create a chance for operation. Recently, *EGFR/BRAF*‐targeted therapy ± chemotherapy has been explored as the frontline approach for *BRAF* V600E mutant mCRC in ANCHOR‐CRC (NCT03693170) and the BREAKWATER trial (NCT01640405).^25^ These results will provide more evidence about first‐line treatment of *BRAF* V600E mutant mCRC.

As a retrospective cohort study, our research had several intrinsic limitations. First, incomplete data collection is non‐negligible in our study. Nearly 20% of patients had unknown tumor differentiation and MMR status. Unfortunately, there were no adequate data for us to validate the prognostic model because of data deficiency in some cases. Second, only a small fraction of mCRC in our institute underwent next‐generation sequencing, which means that the *BRAF* non‐V600E mutation frequency was underestimated in our cohort. Third, the cases enrolled in this study was accumulated in the last 10 years. Constantly changing treatment strategies during this time were unavoidable to cause time‐lag bias for this study. Indeed, the OS of *BRAF* V600E mutant mCRC in this study was longer than previous results published by our team in 2017.^26^ The *EGFR/BRAF*‐targeted therapy has provided more treatment opportunities and has brought about a profound change in the outcome of refractory *BRAF* V600E mutant mCRC in recent years.^27^, ^28^, ^29^ In the future, except for predictors of OS, multiomics models should be developed to identify the optimal treatment (chemotherapy or *EGFR/BRAF*‐targeted therapy) for each patient.

## 5 CONCLUSION

Our study confirmed the prognostic discrepancy in mCRC with different *BRAF* mutations. Patients with the *BRAF* V600E mutation had a significantly unfavorable prognosis in real‐world settings. Meanwhile, intragroup heterogeneity was observed in the specific population. A clinical predictive model based on clinicopathological characteristics could stratify the overall survival of these patients. Additionally, triplet chemotherapy did not significantly improve survival for *BRAF* V600E mutant mCRC patients in real‐world settings.

## AUTHOR CONTRIBUTIONS

**Ting Xu:** Methodology (equal); project administration (equal); writing – original draft (equal). **Jian Li:** Data curation (equal); investigation (equal); methodology (equal). **Zhenghang Wang:** Data curation (equal); software (equal). **Xiaotian Zhang:** Resources (equal); writing – review and editing (equal). **Jun Zhou:** Resources (equal); writing – review and editing (equal). **Zhihao Lu:** Resources (equal); writing – review and editing (equal). **Lin Shen:** Conceptualization (equal); resources (equal); supervision (equal); writing – review and editing (equal). **Xicheng Wang:** Conceptualization (equal); funding acquisition (equal); resources (equal); supervision (equal); writing – review and editing (equal).

## FUNDING INFORMATION

This project is supported by Beijing Xisike Clinical Oncology Research Foundation.

## CONFLICT OF INTEREST STATEMENT

We declare no competing interests.

## ETHICS STATEMENT

The studies involving human participants were reviewed and approved by the Ethics Committee of the Peking University Cancer Hospital. Written informed consent to participate in this study was provided by the participant's legal guardian/next of kin.

## Supporting information


Figure S1.
Table S1.

## Data Availability

The data that support the findings of this study are available from the corresponding author upon reasonable request.
