# Supplementary material for: Real‐world treatment and outcomes of patients with metastatic BRAF mutant colorectal cancer
Source: Cancer Med. 2023 Mar 13;12(9):10473–84. doi: 10.1002/cam4.5783 (PMC10225206; doi:10.1002/cam4.5783)
Supplement: Supplementary file 1 — Figure S1. Table S1. [file CAM4-12-10473-s001.docx]

Table S1 First-line treatment regimens of BRAF V600E mutant mCRC

| Regimen | Number(ratio) |
| --- | --- |
| Fluorouracil/capecitabine | 2 (0.9%) |
| Doublet chemotherapy  oxaliplatin-based with bevacizumab  oxaliplatin-based without bevacizumab  Irinotecan-based with bevacizumab  Irinotecan-based without bevacizumab | 66 (30.7%)  59 (27.4%)  19 (8.8%)  8 (3.7%) |
| Triplet chemotherapy  with bevacizumab  without bevacizumab | 30 (14.0%)  22 (10.2%) |
| Anti-EGFR/BRAF ±MEK inhibitors | 10 (3.7%) |
| Immunotherapy | 1 (0.5%) |


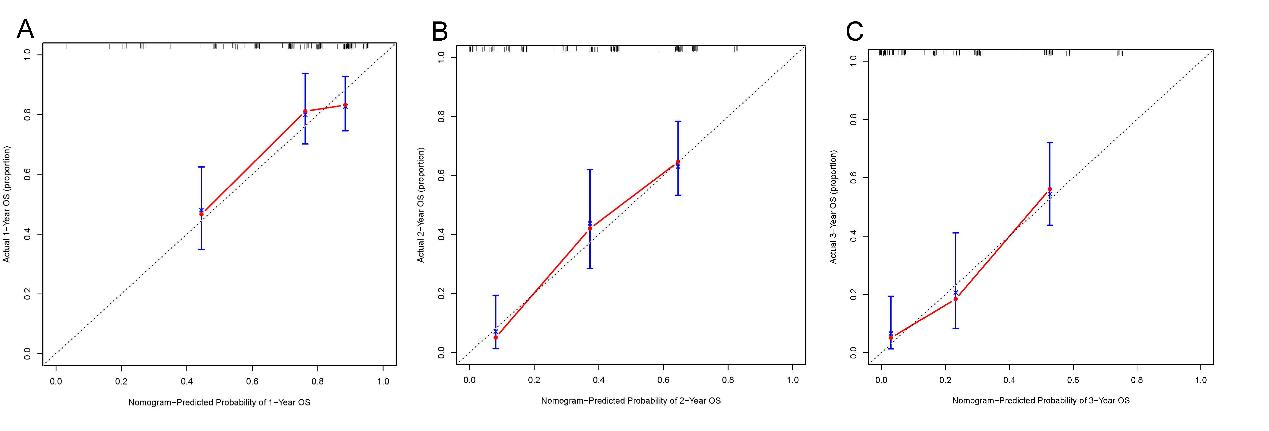


Figure S1 The calibration plots for internal validation the 12-month (A), 24-month (B), 36-month (C) for the nomogram model.
